# Supplementary material for: Immuno-metabolic dendritic cell vaccine signatures associate with overall survival in vaccinated melanoma patients
Source: Nat Commun. 2023 Nov 8;14:7211. doi: 10.1038/s41467-023-42881-4 (PMC10632482; doi:10.1038/s41467-023-42881-4)
Supplement: Supplementary file 1 — Supplementary Information [file 41467_2023_42881_MOESM1_ESM.pdf]

# Supplemental Figure 1

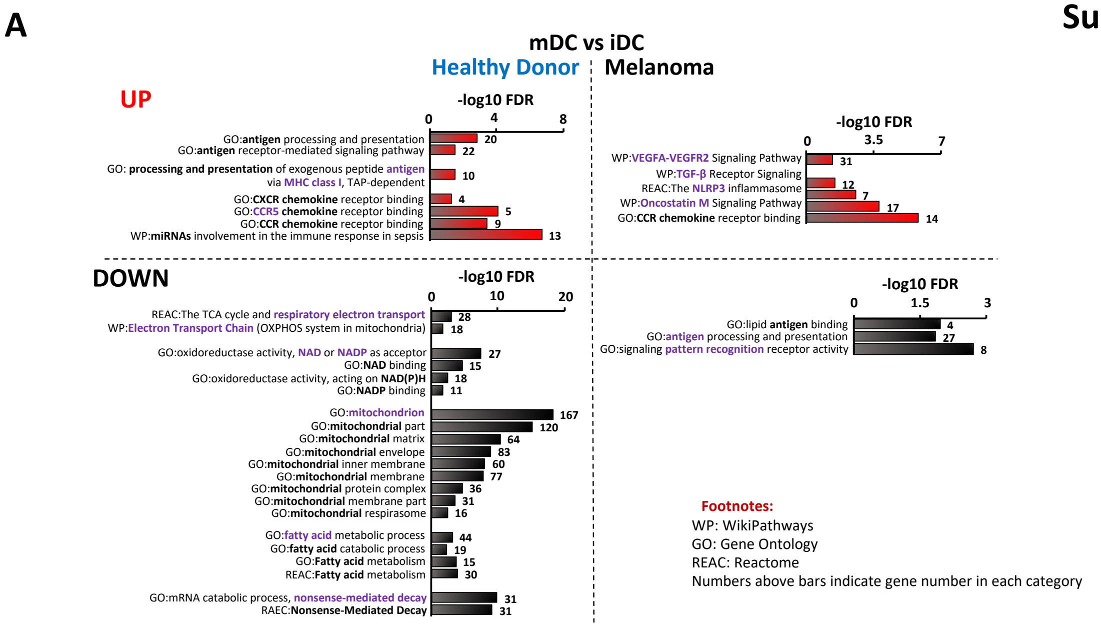


DC maturation protocol comparisons:

**HEALTHY DONOR DCs**

DE genes derived from GEO data repository (GSE111581).
*Jin et al., JTM 2010*
- IL4+GM-CSF = **3** day iDC
- LPS+IFNγ = 24h mDCs

**MELANOMA DCs**

DE genes derived from transcriptional profiling of patient DCs form **09-021 trial**
*Butterfield LH et al., JITC 2019*
- IL4+GM-CSF = **5** day iDC
- LPS+IFNγ = 24h mDCs
- AdVTMM2 = 24h transduced w/ vaccine

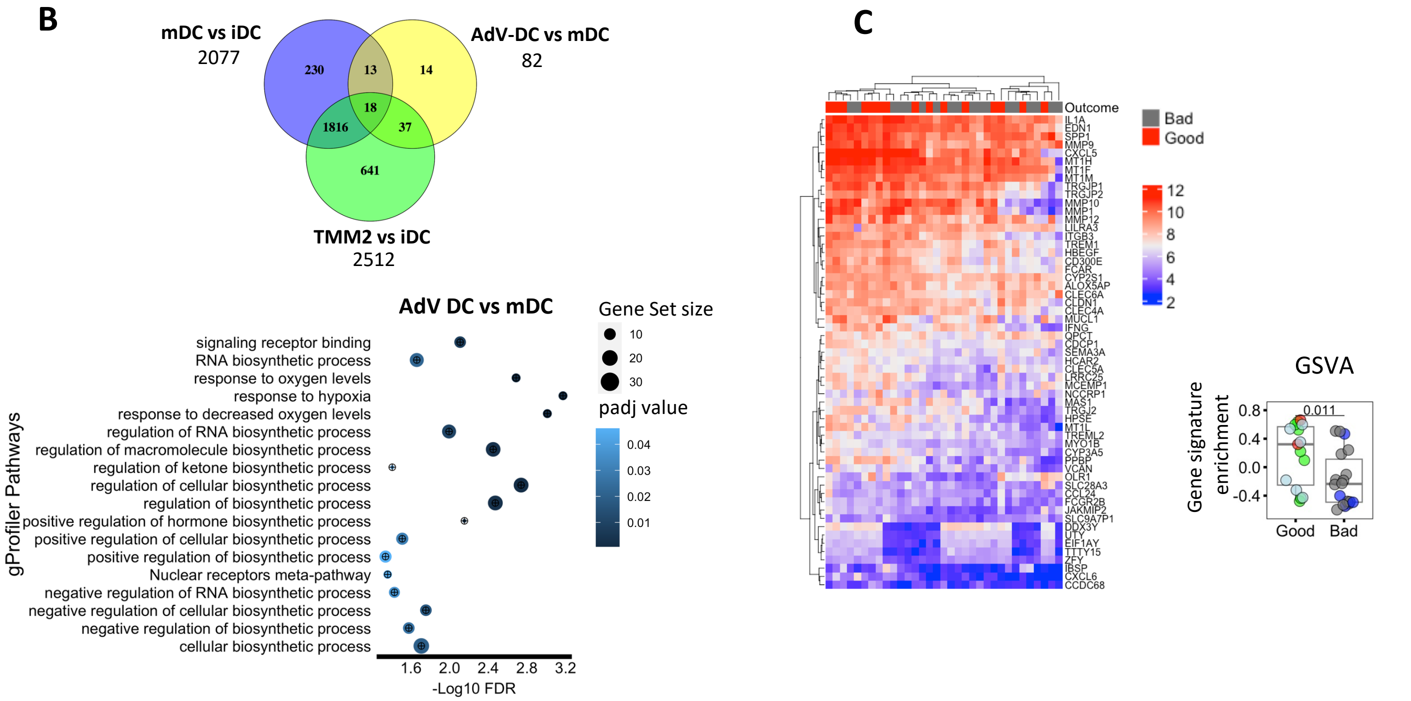


**Supplemental Figure 1. Pathway analysis from DEGs between HD and melanoma patients mDC**

**(A)** G:Profiler was used for pathway enrichment analysis in HD (left) and melanoma mDC (right). Upregulated (Red) and downregulated (Black) biological pathways associated with iDC to mDC maturation are represented. False discovery rate (-log10(FDR)) was used to denote significance for pathway enrichment. Pathway meta-databases used in the analyses are indicated in footnotes. Comparisons of *ex-vivo* DC maturation protocols between the HD-derived (Jin et.al., 2010) (n = 4) and the melanoma (n = 35) mDCs are described. **(B)** Summary of significantly upregulated and downregulated pathways (adj.p<0.05) with overlapping gene sets identified by GSEA/MSigDB analysis between melanoma patient adenovirally engineered AdV-DC vs mDC (n = 35). Dot plot represents summary of significantly (adj.p<0.05) different GSEA/MSigDB pathways between AdV-DC vs mDC. The color-coding scale denotes pathway significance and size of the circles represents the size of leading-edge genes in enriched pathways. **(C)** Heatmap of normalized expression profile for 57 genes that were significantly different between good (PR/SD/NED1, n=15) and bad (PD/NED2, n=18) outcome mDC. Box plot underneath the heatmap represents GSVA analysis for differential enrichment of 57 gene signature between the good and bad outcome groups.

# Supplemental Figure 2

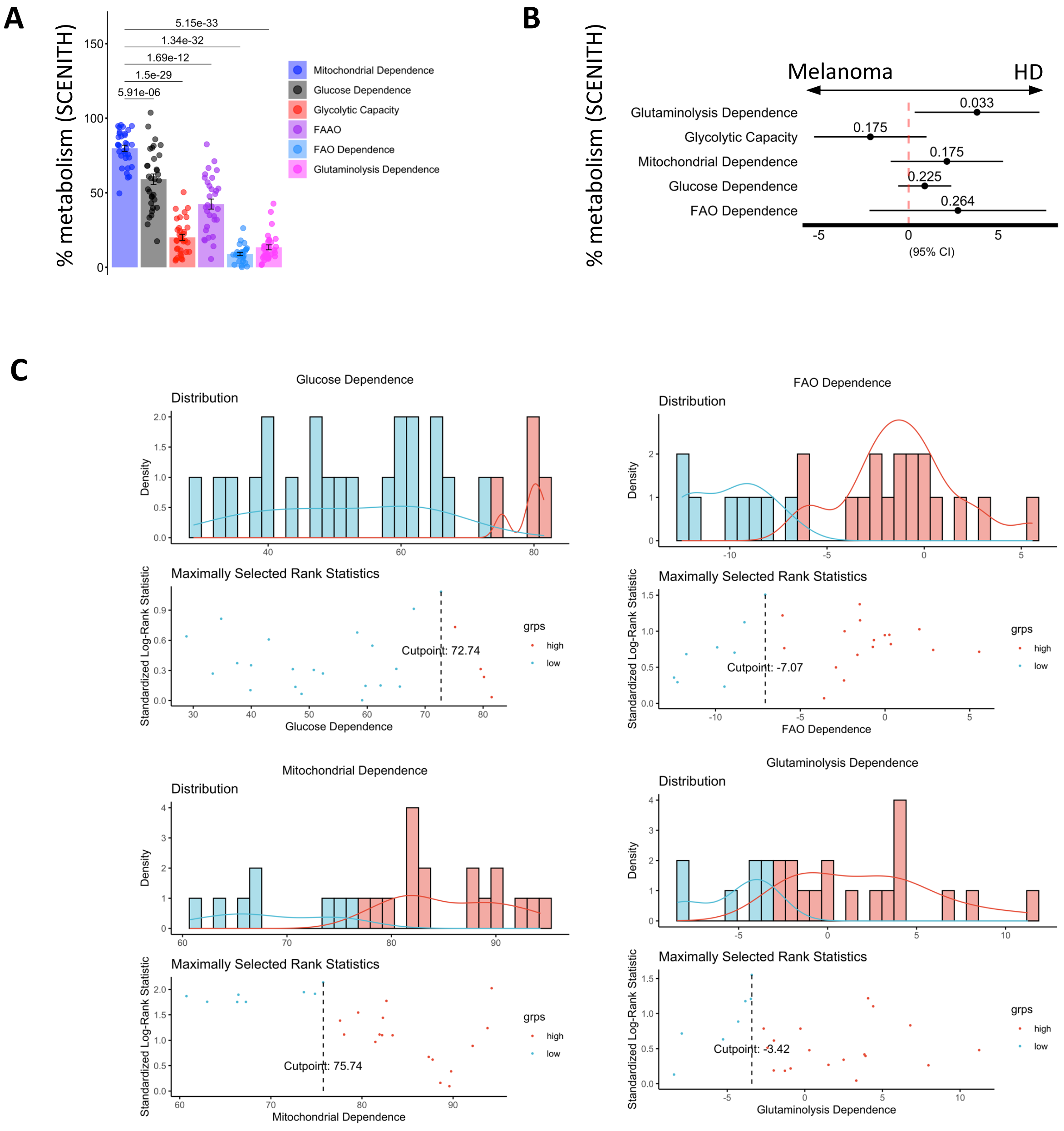


**Supplemental Figure 2. Effects of mDC metabolism on clinical outcome in melanoma patients**
**(A)** Bar graphs represent SCENITH metabolic percentual parameters with dots indicating individual patient revealing the sample distribution. Pairwise comparisons against a HD reference group in were calculated using Two-tailed Student's t-test with Holm-Bonferroni correction (n = 33).
**(B)** Forest plots indicate linear regression results for SCENITH metabolic parameter associations with (HD, n=3), good (PR/SD/NED1, n=13) and bad (PD/NED2, n=17) outcome groups. **(C)** Bag graphs represent distribution density with points denoting maximally selected rank statistics with indicated optimal cutoff value separating high and low SCENITH metabolic parameters with respect to most significant relation to outcome. The indicated cutoff points were used to assess the effect of high/low metabolic parameters in Cox regression and Kaplan-Meier survival analyses of OS and PFS in figures 3B-C. Source data are provided as a Source Data file.

## Supplemental Figure 3

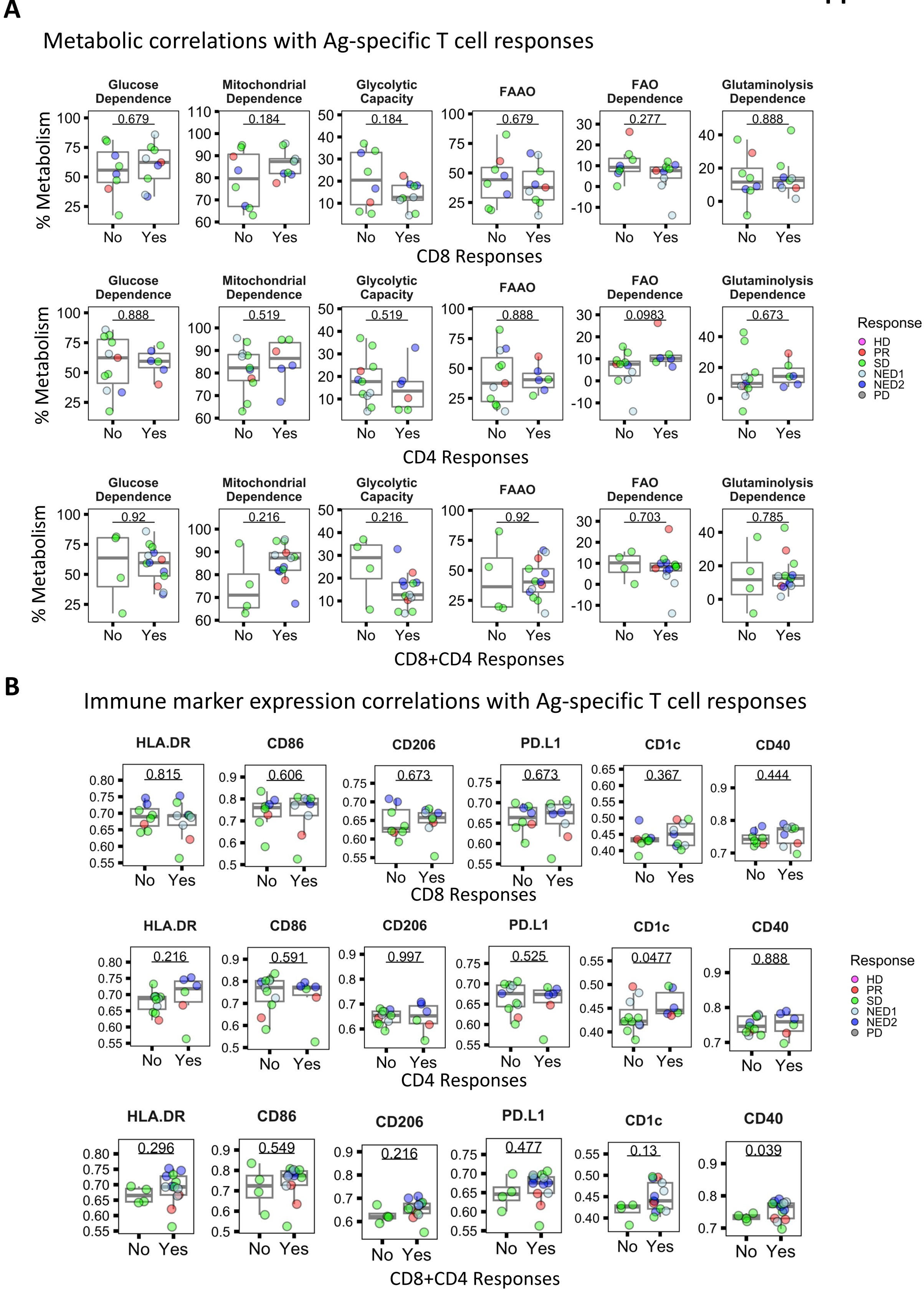


**Supplemental Figure 3. Effects of melanoma mDC metabolism and immune phenotype on T cell responses and clinical response**
**(A)** SCENITH percentual metabolic parameters and **(B)** SCENITH immune marker expression profiles stratified by absence (No) or presence (Yes) of positive CD8, CD4, combined CD8+CD4 IFN-γ T cell responses specific to melanoma antigens (n = 24). In (A-B) Box plots indicate 1st, 2nd and 3rd quartile; whiskers indicate minimum and maximum. Shapiro-Wilk test was used to assess data normality, Two-tailed Wilcoxon signed-rank test (non-normal data) and Two-tailed Student's t-test (normal data) was used for statistical analysis. Source data are provided as a Source Data file.

**Supplemental Figure 4**

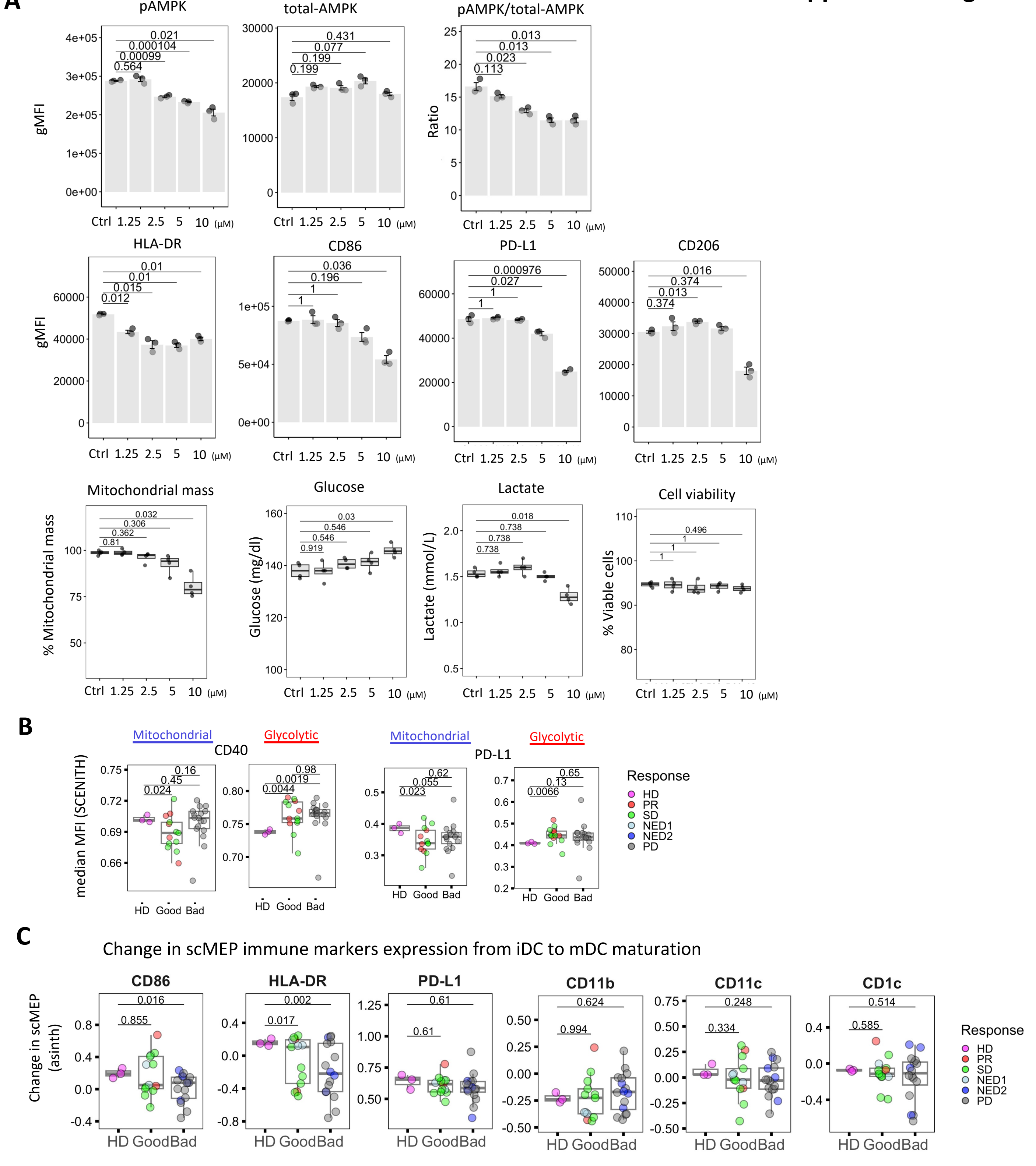


**Supplemental Figure 4. Inhibition of p-AMPK & mitochondrial metabolism impacts immune phenotype of HD DC.**
**(A)** Bar graphs with mean±SE represent gMFI expression values for SCENITH signaling, immune DC markers and the p-AMPK:total-AMPK ratio (N = 3), % mitochondrial mass, glucose, lactate and % viable cells (N = 4) from HD mDC treated with Vehicle (DMSO), or indicated concentrations of Dorsomorphin. Pairwise comparisons against a HD reference group in were calculated using Two-tailed Student's t-test with Holm-Bonferroni correction.
**(B)** Box plots represent differences in expression of median scMEP expression profiles for metabolic markers in mDC between heathy donor (HD, n=3), good (PR/SD/NED1, n=13) and bad (PD/NED2, n=17) clinical groups. **(C)** Box plots represent the change in median scMEP immune marker expression (mDC – iDC) in mDC between heathy donor (HD, n=3), good (PR/SD/NED1, n=13) and bad (PD/NED2, n=17) clinical groups. In (B-C) Box plots indicate 1st, 2nd and 3rd quartile; whiskers indicate minimum and maximum. Source Data file is provided.

## Supplemental Figure 5

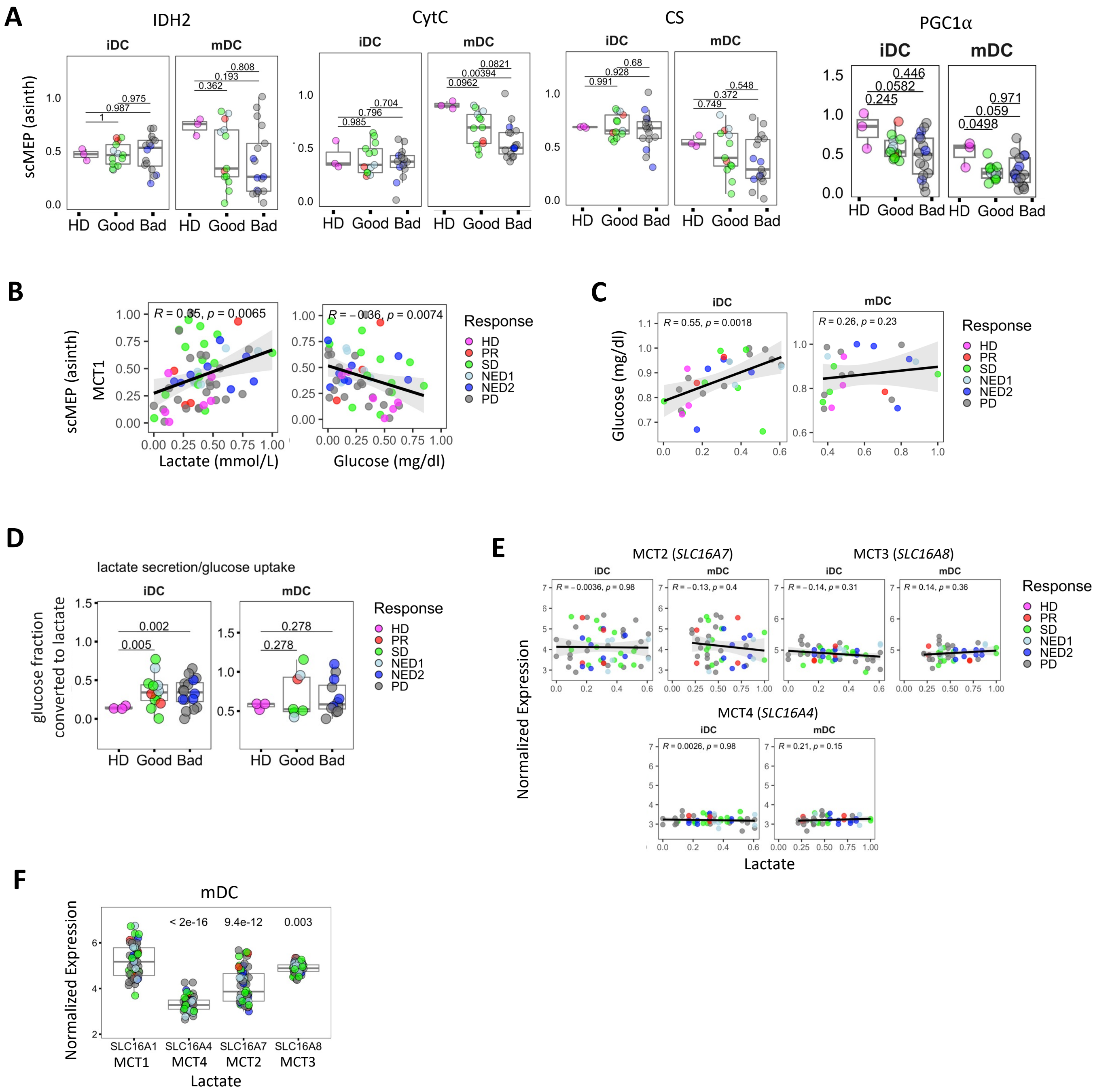


**Supplemental Figure 5. mDC metabolic states, scMEP TCA/ETC marker profiling and lactate and glucose supernatant measurements in HD and melanoma patients**

**(A)** Box plots represent the median scMEP metabolic marker expression in mDC between heathy donor (HD, n=3), good (PR/SD/NED1, n=13) and bad (PD/NED2, n=17) clinical groups.

**(B)** Scatter plots showing correlations between scMEP marker MCT1 and lactate and glucose supernatant measurements. iDC and mDCs values were combined for this analysis (n = 56). **(C)** Scatter plots showing correlations between and lactate and glucose measurements in iDC and mDC supernatants (n = 28). **(D)** Glucose fraction converted to lactate by calculating lactate secretion/glucose uptake in mDC between heathy donor (HD, n=3), good (PR/SD/NED1, n=13) and bad (PD/NED2, n=17) clinical groups. **(E)** Scatter plots showing correlations between lactate supernatant measurements and normalized expression values for MCT2 (*SLC16A7*), MCT3 (*SLC16A8*), and MCT4 (*SLC16A4*) genes from microarray profiling. iDC (n = 35) and mDCs (n = 35) values were combined for this analysis. **(F)** Comparative profiles of normalized gene expression for MCT family of lactate transporters in melanoma mDC (n = 35). In (A, D, F) Box plots indicate 1$^{st}$, 2$^{nd}$ and 3$^{rd}$ quartile; whiskers indicate minimum and maximum. In **(B, C, E)** Spearman correlation coefficient (R), p-values based on asymptotic *t* approximation and 95% confidence intervals in grey shading are indicated. Source data are provided as a Source Data file.

# Supplemental Figure 6

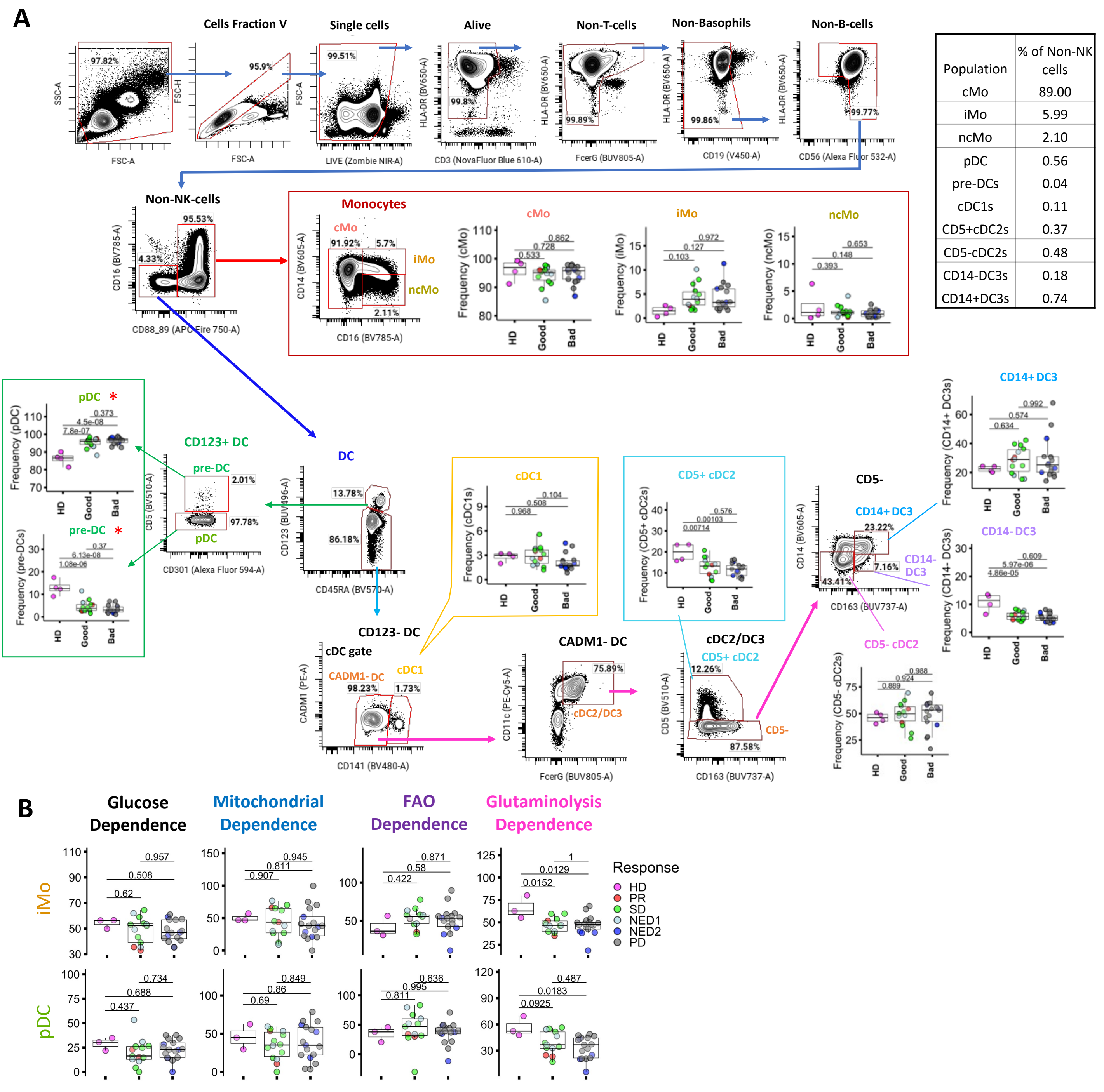


| Population | % of Non-NK cells |
|---|---|
| cMo | 89.00 |
| iMo | 5.99 |
| ncMo | 2.10 |
| pDC | 0.56 |
| pre-DCs | 0.04 |
| cDC1s | 0.11 |
| CD5+cDC2s | 0.37 |
| CD5-cDC2s | 0.48 |
| CD14-DC3s | 0.18 |
| CD14+DC3s | 0.74 |

**Supplemental Figure 6. Fraction V Gating Strategy and population Frequency Data**

**(A)** Overview of gating strategy for circulating melanoma-patient derived monocytes and dendritic cells profiled in this study with table indicating % frequencies of cells in total non-NK gated population. Monocyte fractions obtained from elutriated leukapheresis from melanoma patients (n=30) were analyzed using 31-color spectral flow cytometry panel (Supplemental Table 3) to delineate DC and monocyte subsets. The lineage markers CD3, FcεRG, CD19 and CD56 were used to exclude T, basophils, B cells and NK cells based on lack of HLA-DR. DCs were selected as $CD88^{-}CD89^{-}$ (dual labels on same channel) and $CD16^{-}$ cells from the $HLA\text{-}DR^{+}$ fraction. Monocyte subsets as defined based on CD14 and/or CD16 expression included classical (cMo=$CD14^{high}$), intermediate (iMo= $CD14^{high,}$ $CD16^{high}$) and nonclassical (ncMo= $CD14^{low,}$ $CD16^{high}$) monocytes. $HLA\text{-}DR^{+}CD88^{-}CD89^{-}$ cells were further used to profiled DC subsets. pDCs are $CD123^{+}$ and pre-DCs are $CD123^{+}$ as well as $CD5^{+}CD301^{+}$. $CD123^{-}$ DCs were subdivided into $CADM1^{+}CD141^{+}$ cDC1s and $Fc\varepsilon RG^{+}CD11c^{+}$ cDC2s. The latter was further separated into $CD5^{+}CD163^{-}$ cDC2s, CD5-$CD163^{-}CD14^{-}$ cDC2s, CD5-$CD163^{+}CD14^{-}$ DC3s and CD5-$CD163^{+}CD14^{+}$ DC3s subsets. Box plots represent median population frequencies changes between (HD, n=4), good (PR/SD/NED1, n=13) and bad (PD/NED2, n=17) outcome categories. **(B)** Box plots represent differences in SCENITH metabolic parameters in circulating iMo and pDC between heathy donor (HD, n=3) vs. good (PR/SD/NED1, n=13) and bad (PD/NED2, n=17) response groups. In (A-B) Box plots indicate 1st, 2nd and 3rd quartile; whiskers indicate minimum and maximum. Pairwise comparisons against a HD reference group in were calculated using Two-tailed Student's t-test with Holm-Bonferroni correction. Source data are provided as a Source Data file.

# Supplemental Figure 7

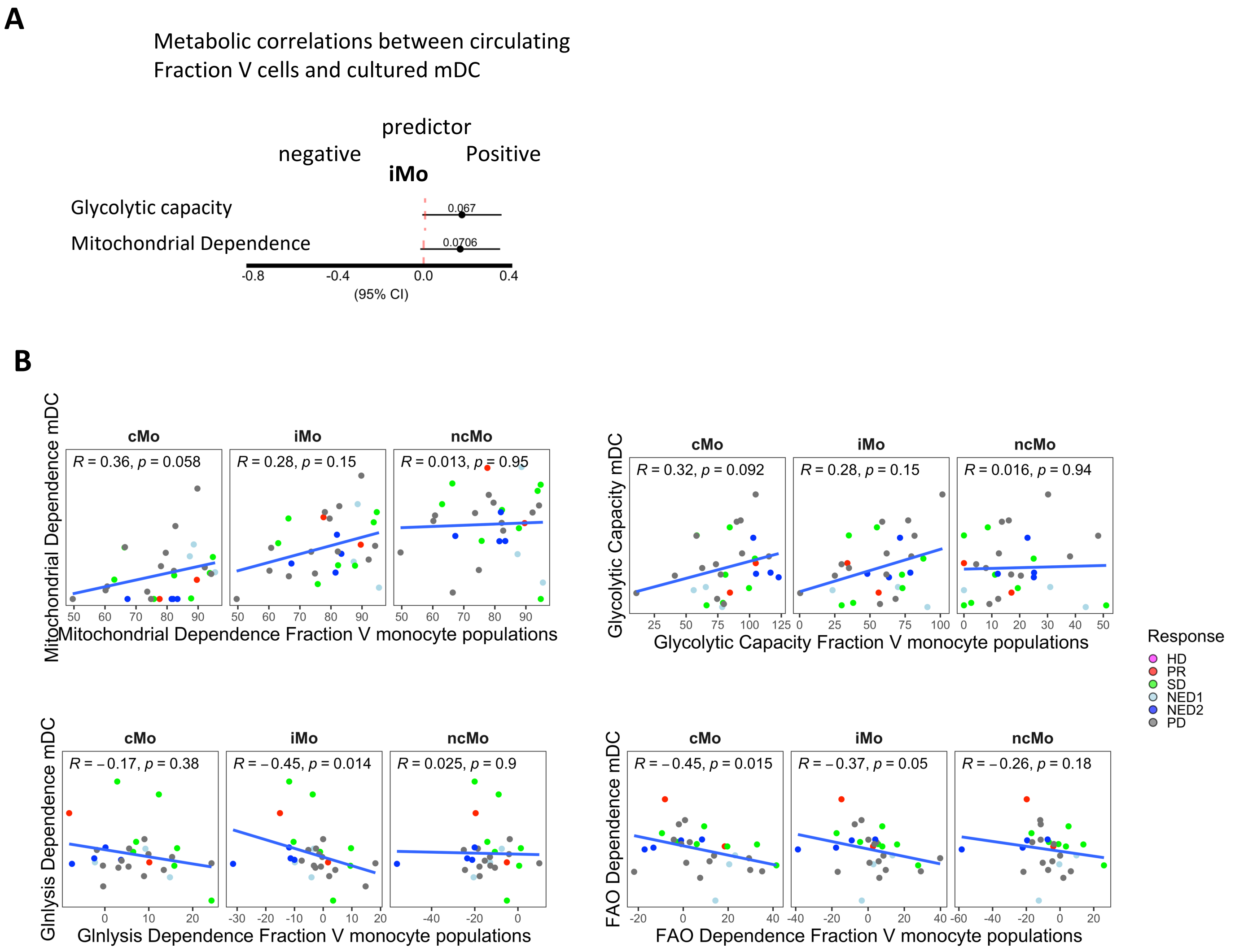


**Supplemental Figure 7. Correlations between metabolic profiles of circulating iMo and cultured mDC from melanoma patients**
**(A)** Forest plot summarizing linear regression associations for SCENITH metabolic parameters glycolytic capacity and mitochondrial dependence between circulating iMo and cultured mDC from melanoma patients. P-values and 95% confidence intervals indicated (n = 30). **(B)** Scatter plots showing correlations between SCENITH percentual parameters between Fraction V circulating monocytic precursors (cMo, iMo, ncMo) and cultured mDC from melanoma patients, p-values based on asymptotic *t* approximation and 95% confidence intervals in grey shading are indicated (n = 30). Source data are provided as a Source Data file.

Supplemental Table 1. Antibodies for flow cytometry and SCENITH

| Marker | Fluorophore | Company | Catalogue # | Clone | Host |
|---|---|---|---|---|---|
| HLA-DR | BV650 | Bioloegend | 307649 | L243 | Mouse |
| LIVE | Zombie NIR-A | BioLegend | 423105 | NA | NA |
| CD98 | BUV496 | BD | 750701 | UM7F8 | Mouse |
| CD36 | BUV395 | BD | 565422 | CB38 | Mouse |
| CD141 | BV480 | BD | 746604 | 1A4 | Mouse |
| total mTOR | PE | BD | 563489 | O21-404 | Mouse |
| CD40 | BV510 | Bioloegend | 334330 | 53C | Mouse |
| CD1c | BV421 | Bioloegend | 331526 | L161 | Mouse |
| Puromycin | Alexa Fluor 488 | Millipore Sigma | MABE343-AF488 | 12D10 | Mouse |
| CD303 | BUV615 | BD | 751078 | V24-785 | Mouse |
| PPARg | AF594 | Bioss Inc | bs-4590R-A594 | NA | Rat |
| PD-L1 | BUV563 | BD | 741423 | MIH1 | Mouse |
| ILT3 | BB700 | BD | 746130 | ZM3.8 | Mouse |
| CD206 | BV711 | Bioloegend | 321135 | 15.2 | Mouse |
| ICOSLG | BV750 | BD | 746899 | 2D3/B7-H2 | Mouse |
| CD86 | R718 | BD | 567071 | 2331 | Mouse |
| p-AMPK | Alexa Fluor 647 | Bioss Inc | bs-4002R-A647 | NA | Rat |
| p-mTOR | PE-Cy7 | eBioscience/TF | 50-112-3458 | MRRBY | Mouse |
| total AMPK | Dyligtht 350 | Novus Bio | NBP2-22127UV | 2B7 | Mouse |

| Dilution |
| --- |
| 5x |
| 10x |
| 5x |
| 5x |
| 5x |
| 5x |
| 5x |
| 5x |
| 10x |
| 5x |
| 2.5x |
| 5x |
| 5x |
| 5x |
| 5x |
| 5x |
| 2.5x |
| 2.5x |
| 2.5x |

Supplemental Table 2: Antibodies for scMEP

| Metal | Myeloid/DC | clone | Column2 | Group |
|---|---|---|---|---|
| 89 | CD45 | HI30 | surface | DC |
| 113 | CD11c | Bu15 | surface | DC |
| 115 | CD11b | ICRF44 | surface | DC |
| 127 | IdU | | other | DNA_RNA_PROT |
| 140 | CD3 | UCHT1 | surface | DC |
| 141 | CD98 | UM7F8 | surface | AA |
| 142 | HADHA | EPR17940 | intracellular | FAO |
| 143 | GSS | EPR6563 | intracellular | ROS |
| 144 | XBP1 | polyclonal | intracellular | SIGNAL |
| 145 | GLS | polyclonal | intracellular | AA |
| 146 | ATF4 | EPR18111 | intracellular | SIGNAL |
| 147 | GAPDH | 6C5 | intracellular | GLYC |
| 148 | CD14 | RMO52 | surface | DC |
| 149 | CytC | 6H2.B4 | intracellular | ETC_TCA |
| 150 | SDHA | 2E3GC12FB2AE2 | intracellular | ETC_TCA |
| 151 | Puromycin | R4743L-E8 | intracellular | DNA_RNA_PROT |
| 152 | ENO1 | EPR10863(B) | intracellular | GLYC |
| 153 | CS | EPR8067 | intracellular | ETC_TCA |
| 154 | BrU | 3D4 | intracellular | DNA_RNA_PROT |
| 155 | CD163 | GHI/61 | surface | HEME |
| 156 | PFKFB4 | polyclonal | intracellular | GLYC |
| 157 | PDK1 | 2H3AA11 | intracellular | SIGNAL |
| 158 | ATP5A | 15H4C4 | intracellular | ETC_TCA |
| 159 | CD86 | IT2.2 | surface | DC |
| 161 | TOMM20 | EPR15581-54 | intracellular | MITO |
| 162 | G6PD | EPR20668 | intracellular | PPP |
| 163 | CD36 | 5-271 | surface | FAO |
| 164 | CD1c | L161 | surface | DC |
| 165 | PGC1a | polyclonal | intracellular | MITO |
| 166 | GLUT1 | EPR3915 | surface | GLYC |
| 167 | CD303 | 201A | surface | DC |
| 168 | CD206 | 15-2 | surface | DC |
| 169 | LDHA | EP1566Y | intracellular | GLYC |
| 170 | IDH2 | EPR7577 | intracellular | ETC_TCA |
| 171 | HK2 | 3D3 | intracellular | GLYC |
| 172 | MCT1 | P14612 | surface | GLYC |
| 173 | CPT1A | 8F6AE9 | intracellular | FAO |
| 174 | ASCT2 | CAL33 | surface | AA |
| 175 | PDL1 | 29E.2A3 | surface | DC |
| 176 | HIF1A | EP1215Y | intracellular | SIGNAL |
| 196 | S6_p | A17020B | intracellular | SIGNAL |
| 198 | dead | | | |
| 209 | HLA-DR | L243 | surface | DC |

Supplementary Table 3: Antibodies used for SCENITH

| Marker | Fluorophore | Company | Catalogue Number | Ab Clone | Species |
|---|---|---|---|---|---|
| CD45 | BUV395 | BD | 563792 | IV N816 | Mouse |
| CD303 (BDCA | BUV615 | BD | 751078 | V24-785 | Mouse |
| PD-L1 | BUV563 | BD | 741423 | MIH1 | Mouse |
| FceRI | BUV805 | BD | 749337 | AER-37 | Mouse |
| pAMPK | Alexa Fluor 647 | Bioss Inc | bs-4002R-A647 | NA | Rat |
| pmTOR | PE-Cy7 | eBioscience/TF | 50-112-3458 | MRRBY | Mouse |
| PURO | Alexa Fluor 488 | Millipore Sigma | MABE343-AF488 | 12D10 | Mouse |
| CD14 | BV605 | Bioloegend | 367125 | 63D3 | Mouse |
| CD16 | BV785 | Bioloegend | 302045 | 3G8 | Mouse |
| ICOSLG | BV750 | BD | 746899 | 2D3/B7-H2 | Mouse |
| CD163 | BUV737 | BD | 741863 | VI M38 | Mouse |
| ILT3 | BB700 | BD | 746130 | ZM3.8 | Mouse |
| SynCAM (TSL | PE | MBL Int Corporatior | CM004-5 | 3.00E+01 | Mouse |
| CD11c | PE-Cy5 | BD | 551077 | B-ly6 | Mouse |
| CD206 | BV711 | Bioloegend | 321135 | 15.2 | Mouse |
| HLA-DR | BV650 | Bioloegend | 307649 | L243 | Mouse |
| CD5 | BV510 | Bioloegend | 364018 | L17F12 | Mouse |
| CD141 (BDCA | BV480 | BD | 746604 | 1A4 | Mouse |
| CD3 | NovaFluor Blue 6 | eBioscience/TF | H002T02B05 | UCHT1 | Mouse |
| LIVE | Zombie NIR | Bioloegend | 423105 | NA | NA |
| CD88 | APC-Fire 750 | Bioloegend | 344315 | S5/1 | Mouse |
| CD89 | APC-Fire 750 | Bioloegend | 354115 | A59 | Mouse |
| CD169(Sigleg | Super Bright 436 | eBioscience/TF | 62-1699-41 | 7-239 | Mouse |
| Axl | Super Bright 436 | eBioscience/TF | 62-1087-41 | DS7HAXL | Mouse |
| CD86 | R718 | BD | 567071 | 2331 | Mouse |
| CD56 | Alexa Fluor 532 | Novus Biological | NBP2-47826AF532 | NCAM1/795 | Mouse |
| CD301 | AF594 | R&D | FAB48881T-100UG | | Mouse |
| CD19 | V450 | BD | 560353 | HIB19 | Mouse |
| CD123 | BUV496 | BD | 751836 | 6H6 | Mouse |
| CD45RA | BV570 | Bioloegend | 304132 | HI100 | Mouse |
| CD1c | BV421 | Bioloegend | 331526 | L161 | Mouse |

| Dilution |
|---|
| 5x |
| 5x |
| 5x |
| 5x |
| 2.5x |
| 2.5x |
| 10x |
| 5x |
| 5x |
| 5x |
| 5x |
| 5x |
| 5x |
| 5x |
| 5x |
| 5x |
| 5x |
| 5x |
| 5x |
| 10x |
| 5x |
| 5x |
| 5x |
| 5x |
| 5x |
| 5x |
| 5x |
| 5x |
| 5x |
| 5x |
| 5x |
