# Supplementary material for: Immuno-metabolic dendritic cell vaccine signatures associate with overall survival in vaccinated melanoma patients
Source: Nat Commun. 2023 Nov 8;14:7211. doi: 10.1038/s41467-023-42881-4 (PMC10632482; doi:10.1038/s41467-023-42881-4)
Supplement: Supplementary file 3 — Reporting Summary [file 41467_2023_42881_MOESM3_ESM.pdf]

nature portfolio

Corresponding author(s): Lisa H Butterfield

Last updated by author(s): 09-29-2023

Reporting Summary

Nature Portfolio wishes to improve the reproducibility of the work that we publish. This form provides structure for consistency and transparency in reporting. For further information on Nature Portfolio policies, see our Editorial Policies and the Editorial Policy Checklist.

Statistics

For all statistical analyses, confirm that the following items are present in the figure legend, table legend, main text, or Methods section.

n/a	Confirmed	
☐	☒	The exact sample size (*n*) for each experimental group/condition, given as a discrete number and unit of measurement
☐	☒	A statement on whether measurements were taken from distinct samples or whether the same sample was measured repeatedly
☐	☒	The statistical test(s) used AND whether they are one- or two-sided<br>*Only common tests should be described solely by name; describe more complex techniques in the Methods section.*
☐	☒	A description of all covariates tested
☐	☒	A description of any assumptions or corrections, such as tests of normality and adjustment for multiple comparisons
☐	☒	A full description of the statistical parameters including central tendency (e.g. means) or other basic estimates (e.g. regression coefficient) AND variation (e.g. standard deviation) or associated estimates of uncertainty (e.g. confidence intervals)
☐	☒	For null hypothesis testing, the test statistic (e.g. *F*, *t*, *r*) with confidence intervals, effect sizes, degrees of freedom and *P* value noted<br>*Give P values as exact values whenever suitable.*
☒	☐	For Bayesian analysis, information on the choice of priors and Markov chain Monte Carlo settings
☒	☐	For hierarchical and complex designs, identification of the appropriate level for tests and full reporting of outcomes
☐	☒	Estimates of effect sizes (e.g. Cohen's *d*, Pearson's *r*), indicating how they were calculated

Our web collection on statistics for biologists contains articles on many of the points above.

Software and code

Policy information about availability of computer code

Data collection	FCS data acquisition was performed using the Cytek Aurora flow cytometer using the using the SpectroFlo Software v2.2.0.1. scMEP samples were acquired on a CyTOF2 mass cytometer (Fluidigm).
Data analysis	As detailed in the Materials and Methods section: For the Microarray data analysis, differential gene expression was analyzed using limma (Version 3.38.3) with weights generated by the voom function66,67. A log2 fold change of 2 and FDR-adj.p-value threshold of 0.05 was used to determine statistical significance. Web-based tool gProfiler68 was used for pathway analysis of significantly up and down-regulated gene sets. Gene set enrichment analysis (GSEA) was conducted using gene sets from the Molecular Signature Database (MSigDB, Version 6.2) in the C2 curated gene category (2005, PNAS 102, 15545-15550). Plots were generated using the R package ggplot2 (Version 3.1.1) and the javaGSEA application (version 3.0). Molecular interaction networks were determined and visualized using the Cytoscape (version 3.7.0).<br>For in-vitro cultured mDC, cell gating for both SCENITH and scMEP was performed using the CellEngine (CellCarta) software and CD14-HLA-DR +CD86+ populations were imported into R environment for correlation and heatmap clustering analyses using the below described R packages. For scMEP raw mass spectrometry data were pre-processed, de-barcoded and imported into R environment using the flowCore package (version 2.0.1). Values were arcsinh transformed (cofactor 5) and normalized for downstream analyses. SCENITH and scMEP dimensionality reduction principal component analysis (PCA) and T-distributed stochastic neighbor embedding (tSNE) analyses were performed using stats (version 4.1.3) and Rtsne (version 0.15), respectively. Uniform Manifold Approximation and Projection (UMAP) was performed using R package umap. (version 2.9.0). For visualization and heatmap clustering we utilized R packages ggplot2 (version 3.3.3) and ComplexHeatmap (version 2.4.3)74, respectively. Stats (version 4.1.3) was used for linear regression analyses and Spearman correlation coefficient correlation matrix for marker expression profiles was computed and visualized using the corrr (version 0.4.3), Hmisc (version 4.5.0) and corrplot (version 0.88) R packages.

For manuscripts utilizing custom algorithms or software that are central to the research but not yet described in published literature, software must be made available to editors and reviewers. We strongly encourage code deposition in a community repository (e.g. GitHub). See the Nature Portfolio guidelines for submitting code & software for further information.

Data

Policy information about availability of data

All manuscripts must include a data availability statement. This statement should provide the following information, where applicable:
- Accession codes, unique identifiers, or web links for publicly available datasets
- A description of any restrictions on data availability
- For clinical datasets or third party data, please ensure that the statement adheres to our policy

The datasets generated during and/or analyzed during the current study are available from the corresponding author on reasonable request.The heathy donor microarray publicly available data used in this study are available in the GEO database under accession code GSE111581 https://www.omicsdi.org/dataset/geo/GSE111581. The melanoma patient microarray data generated in this study have been deposited in the GEO database under accession code GSE157738 https://www.ncbi.nlm.nih.gov/geo/query/acc.cgi?acc=GSE157738.

Human research participants

Policy information about studies involving human research participants and Sex and Gender in Research.

Reporting on sex and gender	NA
Population characteristics	NA
Recruitment	NA
Ethics oversight	NA

Note that full information on the approval of the study protocol must also be provided in the manuscript.

Field-specific reporting

Please select the one below that is the best fit for your research. If you are not sure, read the appropriate sections before making your selection.

[x] Life sciences [ ] Behavioural & social sciences [ ] Ecological, evolutionary & environmental sciences

For a reference copy of the document with all sections, see nature.com/documents/nr-reporting-summary-flat.pdf

Life sciences study design

All studies must disclose on these points even when the disclosure is negative.

Sample size	Sample size (35 subjects) was determined based on the Phase I clinical trial patient numbers previously published and cited in the manuscript.
Data exclusions	Data points were only excluded if there was no sample collected or available for particular patient.
Replication	Each subject was treated as an independent experimental/biological replicate. At least 3 healthy donors were used as independent biological replicates for applicable comparative analyses.<br>Technical replicates from multiple donors were conducted to verify assay robustness and data reproducibility.
Randomization	Randomization was not a relevant feature as we were applying a uniform set of techniques across all available samples from the Phase I cohort. Any comparisons groups were determined based on the patient clinical responses or healthy donor status as follows: healthy donor (HD, n=3) vs. good (PR/SD/NED1, n=13) and bad (PD/NED2, n=17)
Blinding	Blinding was not a relevant feature as we were applying a uniform set of techniques across all available samples from the Phase I cohort.

Reporting for specific materials, systems and methods

We require information from authors about some types of materials, experimental systems and methods used in many studies. Here, indicate whether each material, system or method listed is relevant to your study. If you are not sure if a list item applies to your research, read the appropriate section before selecting a response.

Materials & experimental systems

n/a	Involved in the study	
☐	☒	Antibodies
☒	☐	Eukaryotic cell lines
☒	☐	Palaeontology and archaeology
☐	☐	Animals and other organisms
☐	☒	Clinical data
☒	☐	Dual use research of concern

Methods

n/a	Involved in the study	
☒	☐	ChIP-seq
☐	☒	Flow cytometry
☒	☐	MRI-based neuroimaging

Antibodies

Antibodies used	Fluorescently-conjugated antibodies used in the study include: CD14 (BD, 563561), AMPK (Novus Bio, NBP2-22127UV), CD276 (BD, 749897), CD274 (BD, 741423), CD303 (BD, 751078), HLA-DR (BD, 748338), CD1c (BioLeged, 331525), CD80 (BioLeged, 305233), CD141 (BioLeged, 344117), CD206 (BioLeged, 321135), ILT-3 (BD, 747371), CD86 (BioLeged, 305441), anti-Puro (Millipore Sigma, MABE343-AF488), CD11c (Thermo Fisher, A15803), CD273 (BD, 746072), CD36 (BioLeged, 336206), p-mTOR1 (BD, 563489), PPARg (Bioss Inc, bs-4590R-A594), pS6K (NBP2-73209PECY55,Novus Bio), p-mTOR1 (Ser 2448) (eBioscience, 50-112-3458), CD98 (130-126-200, Myltenyi Biotec), p-AMPKa-1/2 (Thr183/Thr172) (Bioss Inc, bs-4002R-A647), GLUT1 (StressMarq, SPC-1295D-APCCY7), FceRI (BD, 749337), CD11c (BD, 551077), CD3 (eBioscience/TF, H002T02B05), CD88 (Bioloegend,344315), CD89 (Bioloegend, 354115) CD56 (Novus Bio, NBP2-47826AF532), CD19 (BD, 560353), CD123 (BD, 751836), CD45RA (Bioloegend, 304132)
Validation	Fluorescently-conjugated antibodies from Biolegend, Novus Bio, Thermo Fisher/eBioscience and Bioss were tested in a variety of assays and each selected antibody clone suitable for FACS recognizing human protein targets was tested on models designed to carefully assess the desired specificity and sensitivity. scMEP antibodies targeting metabolic features were conjugated in-house using an optimized conjugation protocol (Hartmann et al., 2019) and validated on multiple sample types.

Animals and other research organisms

Policy information about studies involving animals; ARRIVE guidelines recommended for reporting animal research, and Sex and Gender in Research

Laboratory animals	NA
Wild animals	NA
Reporting on sex	NA
Field-collected samples	NA
Ethics oversight	NA

Note that full information on the approval of the study protocol must also be provided in the manuscript.

Clinical data

Policy information about clinical studies

All manuscripts should comply with the ICMJEguidelines for publication of clinical research and a completedCONSORT checklist must be included with all submissions.

Clinical trial registration	NCT01622933
Study protocol	https://clinicaltrials.gov/study/NCT01622933
Data collection	https://clinicaltrials.gov/study/NCT01622933
Outcomes	Clinical outcomes for analysis were described in detail previously, briefly: “good” outcomes were PR+SD >6 mo.+ non-recurrent NED that was high risk at study entry (or NED1); “bad” outcomes were PD +SD</=6 mo. + recurrent high risk NED.

Flow Cytometry

Plots

Confirm that:

☒ The axis labels state the marker and fluorochrome used (e.g. CD4-FITC).

☒ The axis scales are clearly visible. Include numbers along axes only for bottom left plot of group (a 'group' is an analysis of identical markers).

☒ All plots are contour plots with outliers or pseudocolor plots.

☒ A numerical value for number of cells or percentage (with statistics) is provided.

Methodology

Sample preparation	Day 5 Immature Dendritic Cells (DC) were generated from cryopreserved elutriated healthy donor and patient monocytes using 1000 U/mL GM-CSF (Genzyme and Sanofi) and IL-4 (Cell Genix). Dendritic Cells were matured using rhIFNγ (1000 U/mL) (Actimmune and R&D Systems)and LPS (250ng) (Sigma Aldrich) in DC medium for 24hrs. Immature and matured Dendritic Cells were harvested. Viability was analyzed using a Trypan Blue viability dye. Total RNA from 5x10e6 iDC, mDC and vaccine DC was isolated using RNAlater (Qiagen). Metabolic assays were performed as described in Santos et. al, 201970. Day5 immature and Day6 matured were plated at 100,000cells/well on Seahorse culture plates. DMEM media was used, supplemented with 1% BSA, 25mM glucose, 1mM pyruvate, and 2mM glutamine. Basal oxygen consumption and extracellular acidification rates were collected every 30 minutes. The cells were stimulated with oligomycin (2 µM), FCCP (0.5 µM), 2-deoxyglucose (10 mM) and rotenone/antimycin A (0.5 µM) to obtain maximal respiratory and control values. Fatty Acid Beta Oxidation was measured using the XF Palmitate Oxidation Stress Test Kit (Aligent). To measure oxidation levels, palmitate-BSA or BSA control (30 uls) was added to the wells immediately prior to running the assay. Cells were stimulated with oligomycin (2 µM), FCCP (0.5 µM), 2-deoxyglucose (10 mM) and rotenone/antimycin A (0.5 µM) to obtain maximal respiratory and control values. For both metabolic assays, the measurements were performed in triplicates.
Instrument	HUGENE 2.0 ST arrays (Affymetrix) was used for gene expression analyses. Metabolic assays were performed using the Seahorse XFe96 (Agilent). SCENITH FCM was performed using Cytek Aurora flow cytometer and scMEP samples were acquired on a CyTOF2 mass cytometer (Fluidigm). The human immune monitoring 65-Plex (Thermo-Fisher Procarta Plex) was used to analyze pro-inflammatory cytokines in cell-free supernatants harvested from HD (n=4) vs. melanoma patient (n=23) mDC. The human Checkpoint 14-plex kit (Thermo-Fisher Procarta Plex) was also used for detection of culture supernatant checkpoint and costimulatory molecules.
Software	SpectroFlo Software v2.2.0.1 was used for SCENITH FCM acquisition and cell gating for both SCENITH and scMEP was performed using the CellEngine (CellCarta) software. Code, data files and workflows generated for these analyses are available in a GitHub repository at https://github.com/ButterfieldLab/
Cell population abundance	Approximately 200,000 cells was collected for each sample analysis.
Gating strategy	FCS/SSC and live/dead staining was used to identify singlet and viable cells, following by utilization of marker combinations for specific cell type population identification.

☒ Tick this box to confirm that a figure exemplifying the gating strategy is provided in the Supplementary Information.
